# Distributed Harmonization: Federated Clustered Batch Effect Adjustment and Generalization

**DOI:** 10.1145/3637528.3671590

**Published:** 2024-08-24

**Authors:** Bao Hoang, Yijiang Pang, Siqi Liang, Liang Zhan, Paul M. Thompson, Jiayu Zhou

**Affiliations:** Michigan State University, East Lansing, Michigan, USA; Michigan State University, East Lansing, Michigan, USA; Michigan State University, East Lansing, Michigan, USA; University of Pittsburgh, Pittsburgh, Pennsylvania, USA; University of Southern California, Los Angeles, California, USA; Michigan State University, East Lansing, Michigan, USA

**Keywords:** Harmonization, Distributed Algorithm, Neuroimaging, Medical Data

## Abstract

Independent and identically distributed (*i.i.d.*) data is essential to many data analysis and modeling techniques. In the medical domain, collecting data from multiple sites or institutions is a common strategy that guarantees sufficient clinical diversity, determined by the decentralized nature of medical data. However, data from various sites are easily biased by the local environment or facilities, thereby violating the *i.i.d.* rule. A common strategy is to harmonize the site bias while retaining important biological information. The COMBAT is among the most popular harmonization approaches and has recently been extended to handle distributed sites. However, when faced with situations involving newly joined sites in training or evaluating data from unknown/unseen sites, COMBAT lacks compatibility and requires retraining with data from all the sites. The retraining leads to significant computational and logistic overhead that is usually prohibitive. In this work, we develop a novel *Cluster ComBat* harmonization algorithm, which leverages cluster patterns of the data in different sites and greatly advances the usability of COMBAT harmonization. We use extensive simulation and real medical imaging data from ADNI to demonstrate the superiority of the proposed approach. Our codes are provided in https://github.com/illidanlab/distributed-cluster-harmonization.

## 1 INTRODUCTION

The recent advances in machine learning approaches have greatly advanced biomedical data analysis. In brain imaging analysis, for example, Magnetic Resonance Imaging (MRI) has been used for the detection and disease progression of many diseases, such as Mild Cognitive Impairment (MCI) [41, 55–57], Parkinson’s disease [12], and Brain Tumor Detection [1]. However, one critical challenge with brain imaging is that the brain imaging is sensitive to scanner or protocol effect [30, 52], also commonly referred to as site effect or batch effect, leading to the fact that brain imaging from multiple sites is not independent and identically distributed (*i.i.d.*). The bias in non-*i.i.d.* data will cause unstable prediction performance and poor generalization performance to unseen data [49]. Consequently, developing an algorithm that can eliminate these types of bias ensures consistent and reliable outcomes in the deployment of machine learning models within the medical imaging domain.

COMBAT [21] is a well-known harmonization technique and has been shown to be helpful in mitigating the site effect of neuroimaging data introduced by multiple sites sampling [28]. Despite its utility, one of the central ideas is that COMBAT debias the site effect independently according to the (local) site data, which induces one critical limitation, its inability to evaluate site effects coming from unseen or unknown sites without undergoing a retraining process. The requirement of retraining is hindered by substantial computational costs when it comes to real-world deployment, especially when dealing with large datasets from multiple sites. This limitation underscores the need for a more efficient and broadly applicable approach in mitigating the site effects of medical data, especially neuroimaging data.

Furthermore, a centralized setting for site effect harmonization introduces extra concerns. For instance, sharing data directly among multiple sites to apply COMBAT harmonization poses challenges to the security of confidential data and the protection of patient privacy. Direct training on all the data is often impractical in the medical domain. This underscores the need to develop harmonization algorithms in a decentralized manner that can effectively harmonize without gathering data from all sites while maintaining competitive performance in the centralized setting. Particularly, the Distributed ComBat [6], a distributed version of the COMBAT harmonization algorithm, has demonstrated its harmonization capability meanwhile obeying decentralized manners. Nevertheless, Distributed ComBat suffers from the same limitation as the original COMBAT, i.e., it cannot estimate site effects from *unseen sites* without retraining.

Because a significant part of site effects in medical data are ultimately rooted in medical instruments, e.g., MRI scanners from different manufacturers and configurations, the bias underneath the sites may not be independent and may exhibit clustering structures. In this paper, we proposed the *Cluster ComBat* method, an extension of the original COMBAT algorithms that leverages the cluster patterns of site effects. This approach enables the estimation of site effects from unknown sites without necessitating a retraining process. Furthermore, we also developed a distributed version *Cluster ComBat* and demonstrated its efficacy in harmonizing data obeying decentralized manners. Our empirical findings show that *Cluster ComBat* in both centralized and decentralized settings outperform their respective counterparts on both synthetic and real-world neuroimaging datasets.

## 2 RELATED WORKS

### Brain Imaging.

The integration of brain imaging and machine learning has drawn significant attention in recent research, with a focus on advancing diagnostic capabilities and understanding complex neurological conditions [26, 37]. Recent research advancements highlight the potential of machine learning techniques in exploring underlying complex patterns within neuroimaging data. For example, T1-weighted MRI with Lasso Regression, a statistical technique [39], has proven effective in detecting MCI [41]. Their findings show promising results in the early detection of MCI, emphasizing the significance of early intervention and treatment. Furthermore, complicated deep learning architectures, such as YOLOv7 [40], have also demonstrated exceptional predictive performance in brain tumor detection using T1-weighted MRI [1]. Besides, Diffusion Tensor Imaging (DTI) also shows valuable information related to Alzheimer’s disease (AD) pathology and achieves promising performance in the diagnosis and progression modeling of AD using machine learning classification [42, 43, 53, 54].

### Distributed Learning.

Preserving the privacy of users’ information is a crucial issue that needs to be considered as an important aspect to evaluate in a learning algorithm [11, 17, 18, 22, 47]. Especially in a healthcare setting, where the health data is sensitive, we need to design a distributed learning approach that avoids leaking any private information from hospitals’ data [14, 44–46]. For example, for brain imaging, federated learning, a distributed machine learning algorithm, has proven to be effective in analyzing neuroimaging for cognitive detection tasks while still protecting patients’ information [8, 33]. Moreover, for health records data, to avoid sharing raw data between sites, the use of first-order and second-order gradients of the likelihood function of sites has been shown to be sufficient for achieving high accuracy in classification tasks [9]. In addition, the distributed version of generalized linear mixed models (GLMM) can also achieve nearly identical results in analyzing electronic health records data as in a centralized setting [48]. Relating to harmonization methods in a decentralized setting, Distributed ComBat [6] and Federated Learning ComBat [36] have been developed to harmonize neuroimaging without the need for sharing information between hospitals.

### ComBat Harmonization.

COMBAT harmonization, initially designed for applications in bioinformatics and genomics, is an effective strategy in mitigating batch effects or site effects within high-dimensional data [21]. It has been adopted to address various situations and problems [2]. Fully Bayesian ComBat [32] investigates the advantage of using Monte Carlo sampling for statistical inference in harmonization algorithms. ComBat-GAM [31] extends the model’s capability by also estimating non-linear effects that came from biological covariates, in contrast to only linear effects considered in the original COMBAT model. Longitudinal ComBat [3] is designed for datasets collected over multiple time points from the same subjects, effectively taking into account variations within each subject over time and considering changes in linear covariates. To preserve the privacy of brain imaging across multiple hospitals or sites, Distributed ComBat [6] introduces a decentralized learning version of the original ComBat, which can also harmonize data in decentralized settings. Combining the strengths of ComBat-GAM and Distributed ComBat, Federated Learning ComBat [36] not only estimates non-linear effects from biological covariates but also utilizes the FedAvg algorithm [25] to protect the privacy of data. However, they are not applicable in large-scale studies when new sites join the analysis after the harmonization (e.g., [34, 38]).

## 3 METHODS

### 3.1 Preliminary: ComBat Harmonization

COMBAT [21] adjusts the location (mean) and scale (variance) of data from different sites for the requirement of downstream analysis tasks. Assume given a dataset with G features collected from M different sites. For each site iϵ[M], there are $N_{i}$ samples, and $N=\sum_{i\in [M]}\;N_{i}$ is the total number of samples. ComBat follows an L/S model assuming that, for each sample $j\in \left[N_{i}\right]$ on site i, the value of gϵ[G] feature $y_{ijg}$ can be modeled as:

(1) $$y_{ijg}=\alpha_{g}+X_{ij}\beta_{g}+\gamma_{ig}+\delta_{ig}\epsilon_{ijg},$$

where $\alpha_{g}$ is the mean value of that feature, $X_{ij}$ is the biological covariates (e.g., age, sex), and $\beta_{g}$ is the regression coefficient of $X_{ij}.\;\gamma_{g}$ represents additive effects from site i, while $\delta_{g}$ represents the corresponding multiplicative effects. Also, the error term $\epsilon_{ijg}$ is assumed to be drawn from a Normal distribution $𝒩\left(0,\sigma_{g}^{2}\right)$. We call these site-wise effect parameters as *harmonization parameters*.

The L/S model assumes that different sites would have different *site effects* on their own data. Thus, removing both additive and multiplicative effects from data within each site is mandatory for the later regression task. The empirical Bayes algorithm is typically used to estimate these harmonization parameters in COMBAT-related approaches [21, 31].

First, COMBAT standardizes the data feature-wise:

(2) $$Z_{ijg}=\frac{y_{ijg}\;-\;{\overset{ˆ}{\alpha }}_{g}\;-\;X_{ij}{\overset{ˆ}{\beta }}_{g}}{{\overset{ˆ}{\sigma }}_{g}},$$

where ${\overset{ˆ}{\sigma }}_{g}^{2}=\frac{1}{N}\sum_{ij}\;{\left(y_{ijg}-{\overset{ˆ}{\alpha }}_{g}-X_{ij}{\overset{ˆ}{\beta }}_{g}-{\overset{ˆ}{\gamma }}_{ig}\right)}^{2}$, and ${\overset{ˆ}{\alpha }}_{g}$, ${\overset{ˆ}{\beta }}_{g}$, ${\overset{ˆ}{\gamma }}_{ig}$ are estimated using feature-wise ordinary least-squares approach.

Then, given distribution assumptions $Z_{ijg}∼𝒩\left(\gamma_{ig},\delta_{ig}^{2}\right)$, $\gamma_{ig}∼𝒩\left(\gamma_{i},\tau_{i}^{2}\right)$, and $\delta_{ig}^{2}∼InverseGamma(\lambda_{i},\theta_{i})$, using empirical Bayes algorithm, we can estimate $\gamma_{ig}^{*}$ and ${\delta_{ig}^{*}}^{2}$ iteratively through:

(3) $$\gamma_{ig}^{*}=\frac{N_{i}{\overset{‾}{\tau }}_{i}^{2}{\overset{ˆ}{\gamma }}_{ig}\;+\;{\delta_{ig}^{*}}^{2}{\overset{‾}{\gamma }}_{i}}{N_{i}{\overset{‾}{\tau }}_{i}^{2}\;+\;{\delta_{ig}^{*}}^{2}},\;{\delta_{ig}^{*}}^{2}=\frac{{\overset{‾}{\theta }}_{i}\;+\;\frac{1}{2}\sum_{j}\;\;{\left(Z_{ijg}\;-\;\gamma_{ig}^{*}\right)}^{2}}{\frac{1}{2}N_{i}\;+\;{\overset{‾}{\lambda }}_{i}-1},$$

where ${\overset{‾}{\tau }}_{i}^{2}$, ${\overset{‾}{\gamma }}_{i}$, $\bar{\theta_{i}}$, $\bar{\lambda_{i}}$ are computed through the method of moments. Finally, harmonized data is obtained within each site using:

(4) $$y_{ijg}^{*}=\frac{{\overset{ˆ}{\sigma }}_{g}}{\delta_{ig}^{*}}\left(Z_{ijg}-\gamma_{ig}^{*}\right)+{\overset{ˆ}{\alpha }}_{g}+X_{ij}{\overset{ˆ}{\beta }}_{g}.$$

### 3.2 Cluster ComBat

Though COMBAT has been widely adopted for various analyses [3, 31], it lacks generalization to new sites. When applied to an unseen site, COMBAT requires re-estimating all harmonization parameters based on M+1 sites, which needs to engage all participating sites to coordinate harmonization, which is costly and usually prohibitive. Also, the original COMBAT assumes that scale and mean effects exist within each single site, and each group of harmonization parameters (i.e., $\gamma_{ig}$ and $\delta_{ig}$ with the same i) can only be estimated within each single site of limited sample size, which may lead to suboptimal estimation of harmonization parameters.

Instead of assuming harmonization parameters can only be shared within each single site, we assume that multiple sites can share one group of harmonization parameters. As such, data points from multiple sites sharing the same harmonization parameters can be clustered into one cluster. We thus reformulate the L/S model as:

(5) $$y_{ijg}=\alpha_{g}+X_{ij}\beta_{g}+\gamma_{cg}+\delta_{cg}\epsilon_{ijg},$$

where cϵ[C] represents the cluster index of site i, and there are C clusters in total, where C≤M. Compared with the original version of ComBat, where we need to estimate G·M harmonization parameters, this cluster-based algorithm only requires the estimation of G·C harmonization parameters.

Using cluster-wise shared harmonization parameters, we can generalize knowledge from previous N sites to the new unseen site once we know which cluster each data point from this site belongs to. Additionally, the estimation process of harmonization parameters $\gamma_{cg}$ and $\delta_{cg}$ can benefit from multiple sites’ data points within the same cluster, considering the sample number for estimating each parameter group is enlarged. And we name this algorithm *Cluster ComBat*.

The *Cluster ComBat* algorithm requires the following steps for harmonization: i) sample clustering using *K*-means, based on data points from all sites, to decide data points’ cluster index of each site; ii) feature-wise standardization on all samples using ${\overset{ˆ}{\alpha }}_{g}$, ${\overset{ˆ}{\beta }}_{g}$ and ${\overset{ˆ}{\sigma }}_{g}$ from least-squares; iii) empirical Bayes estimation of the cluster-wise harmonization parameters $\gamma_{cg}$ and $\delta_{cg}$ for each cluster based on sites within cluster cϵ[C], following Equation 3 with replacing $N_{i}$ to the overall sample number in cluster c; iv) harmonization process following Equation 4 with replacing $\gamma_{ig}^{*}$ and $\delta_{ig}^{*}$ to $\gamma_{cg}^{*}$ and $\delta_{cg}^{*}$ respectively. For the cluster index assignment for each training sample, we directly apply sample-wise index assignment using the clustering algorithm, allowing data points at the same site to have different cluster indexes. This is the “privilege” of the centralized setting, as we can access the feature values of all data from all sites, thereby facilitating the determination of the cluster index for each individual data point. This also allows the cluster index of data points to ignore site belonging, considering the reduction of bias not only from sites but also from other potential factors, leading to better handling of data heterogeneity. The complete process is demonstrated in Algorithm 1.

**Algorithm 1:** Centralized Cluster ComBat

**Input:** $y_{ijg}$ - unharmonized data and $X_{ij}$ - biological covariates of sample j from site i
**Output:** ${\overset{ˆ}{\alpha }}_{g}$, ${\overset{ˆ}{\beta }}_{g}$, $\delta_{cg}^{*}$, $\gamma_{cg}^{*}$ - harmonization parameters and k - trained
K-means model
Train K-means model k using $y_{ijg}$
Estimate ${\overset{ˆ}{\alpha }}_{g}$, ${\overset{ˆ}{\beta }}_{g}$, and ${\overset{ˆ}{\gamma }}_{ig}$ using least-square methods
Standardize data via Equation 2
Get cluster index $c=k\left(y_{ij}\right)$ of every $y_{ij}$
Estimate $\delta_{cg}^{*}$, $\gamma_{cg}^{*}$ using $EmpricalBayes\left(Z_{ijg}\right)$ via Equation 3
**return** ${\overset{ˆ}{\alpha }}_{g}$, ${\overset{ˆ}{\beta }}_{g}$, $\delta_{cg}^{*}$, $\gamma_{cg}^{*}$, k

**Algorithm 2:** *Cluster ComBat* for Unseen Site

**Input:** $y_{ijg}$ - unseen site’s data, $X_{ij}$ - biological covariate of new client, previously estimated parameters ${\overset{ˆ}{\alpha }}_{g}$, ${\overset{ˆ}{\beta }}_{g}$, $\delta_{cg}^{*}$, $\gamma_{cg}^{*}$, and trained K-means k
**Output:** $y_{ijg}^{*}$ - harmonized features
Get cluster index $\tilde{c}=k\left(y_{ij}\right)$
Standardized data via Equation 2
**return** $y_{ijg}^{*}=\frac{{\overset{ˆ}{\sigma }}_{g}}{\delta_{\tilde{c}g}^{*}}(Z_{ijg}-\gamma_{\tilde{c}g}^{*})+{\overset{ˆ}{\alpha }}_{g}+X_{ij}{\overset{ˆ}{\beta }}_{g}$

Now, we introduce how proposed *Cluster ComBat* can apply harmonization to the unseen site i∉[M]. We first use the trained K-means to identify the cluster of each data point $y_{ij}$, denoted as $k\left(y_{ij}\right)$. Then, with pre-estimated harmonization parameters $\delta_{cg}^{*},\;\gamma_{cg}^{*}$, we can derive the harmonized features for this unseen site i by

(6) $$y_{ijg}^{*}=\frac{{\overset{ˆ}{\sigma }}_{g}}{\delta_{\tilde{c}g}^{*}}\left(Z_{ijg}-\gamma_{\tilde{c}g}^{*}\right)+{\overset{ˆ}{\alpha }}_{g}+X_{ij}{\overset{ˆ}{\beta }}_{g},$$

where $\tilde{c}=k\left(y_{ij}\right)$. Algorithm 2 describes the procedure when dealing with data from an unseen site in the centralized setting.

### 3.3 Distributed Cluster ComBat

In the real-world scenario, large-scale analyses often involve medical data from multiple institutions (e.g., [34]). The data is often stored in distributed data centers by various data owners, and raw data cannot be transferred to other locations or directly accessed by other institutions (i.e., sites) due to privacy concerns and regulations. Thus, centralized algorithms like COMBAT cannot be directly applied. Though previous work [6] has made an effort to design a distributed version of COMBAT, it would face the same problem as COMBAT when it comes to the unseen new site.

To this end, we propose *Distributed Cluster ComBat*, extending *Cluster ComBat* by enabling its generalization ability to the unseen site, attributed to the cluster-wise harmonization model. However, unlike sample-feature clustering in a centralized setting as shown in Section 3.2, we perform clustering on locally estimated feature-wise parameters, e.g., $\alpha_{ig}$, $\beta_{ig}$, and $\gamma_{ig}$, to tackle the inaccessibility of raw samples on other sites. The intuition is that if the feature data of sites exhibit a cluster pattern, locally estimated feature-wise parameters will also exhibit the same cluster pattern, which is validated through our simulation studies. Also, the clustering cost is reduced significantly in the distributed version, considering clustering only on M parameter vectors with M≪N.

**Figure 1:**
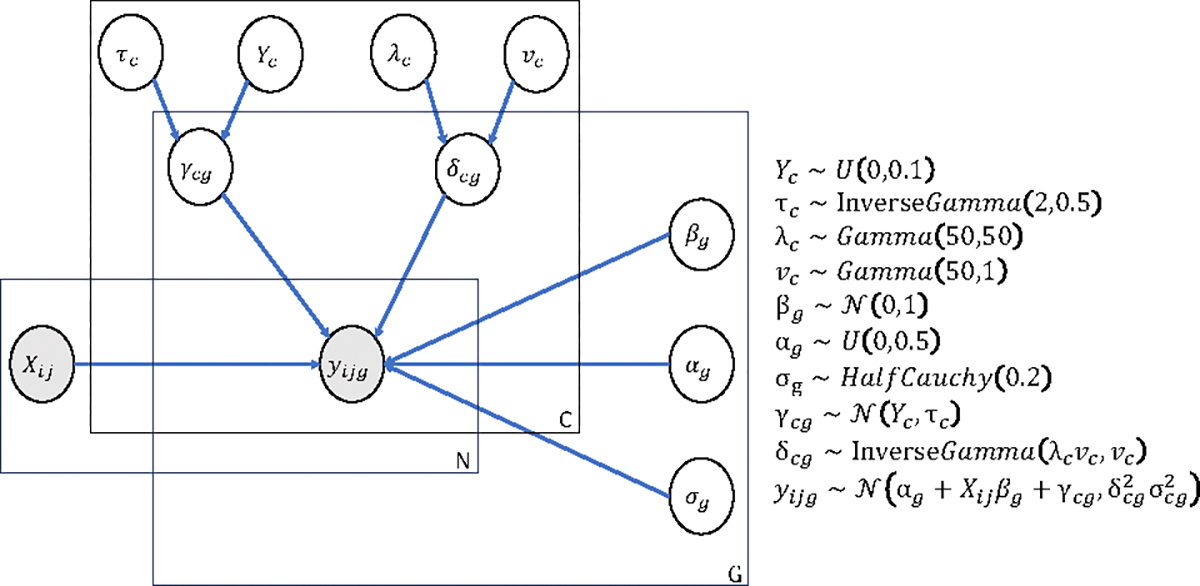
Graphical model used to generate synthetic data. The shaded circles represent observed variables, including biological covariates and feature values, while unshaded circles represent latent parameters.

The *Distributed Cluster ComBat* has the following steps: i) each site estimates feature-wise parameters ${\overset{ˆ}{\alpha }}_{ig}$, ${\overset{ˆ}{\beta }}_{ig}$, and ${\overset{ˆ}{\gamma }}_{ig}$ locally at the same time, and sends parameters to the central server; ii) the central server performs K-means clustering based on ${\overset{ˆ}{\alpha }}_{ig}$, ${\overset{ˆ}{\beta }}_{ig}$, and ${\overset{ˆ}{\gamma }}_{ig}$ for i∈[M]; iii) the central server aggregates ${\left\{{\overset{ˆ}{\alpha }}_{ig}\right\}}_{i\in M}$, ${\left\{{\overset{ˆ}{\beta }}_{ig}\right\}}_{i\in M}$ and ${\left\{{\overset{ˆ}{\gamma }}_{ig}\right\}}_{i\in M}$ to estimates the global feature-wise parameters ${\overset{ˆ}{\alpha }}_{g},\;{\overset{ˆ}{\beta }}_{g}$ and ${\overset{ˆ}{\gamma }}_{ig}$, and then sends back to each site; iv) each site standardized the local data using global feature-wise parameters, then locally estimates ${\overset{ˆ}{\delta }}_{ig}$ and ${\overset{ˆ}{\gamma }}_{ig}$; v) each site sends locally estimated harmonization parameters to the server; vi) server aggregates harmonization parameters within each cluster to estimate the cluster-wise ones, then sends back to each site; the aggregation procedure precisely follows the procedure outlined in Figure 1 of the original Distributed ComBat algorithm paper [6]; vii) each site performs local harmonization based on cluster-wise harmonization parameters. The procedure is summarized in Algorithm 3.

When generalized to new unseen site i∉[M], we first estimate the local feature-wise parameters $\alpha_{ig}$, $\beta_{ig}$ and $\gamma_{ig}$, then use previous trained K-means model k to find the cluster index of the current site based on local estimated feature-wise parameters. Others follow a similar procedure as *Cluster ComBat*, as summarized in Algorithm 4.

## 4 VALIDATION USING SIMULATION

### In simulation, we use controllable synthetic data to validate the correctness of the proposed algorithms and the intuitions used.

### 4.1 Synthetic data generation

**Figure 2:**
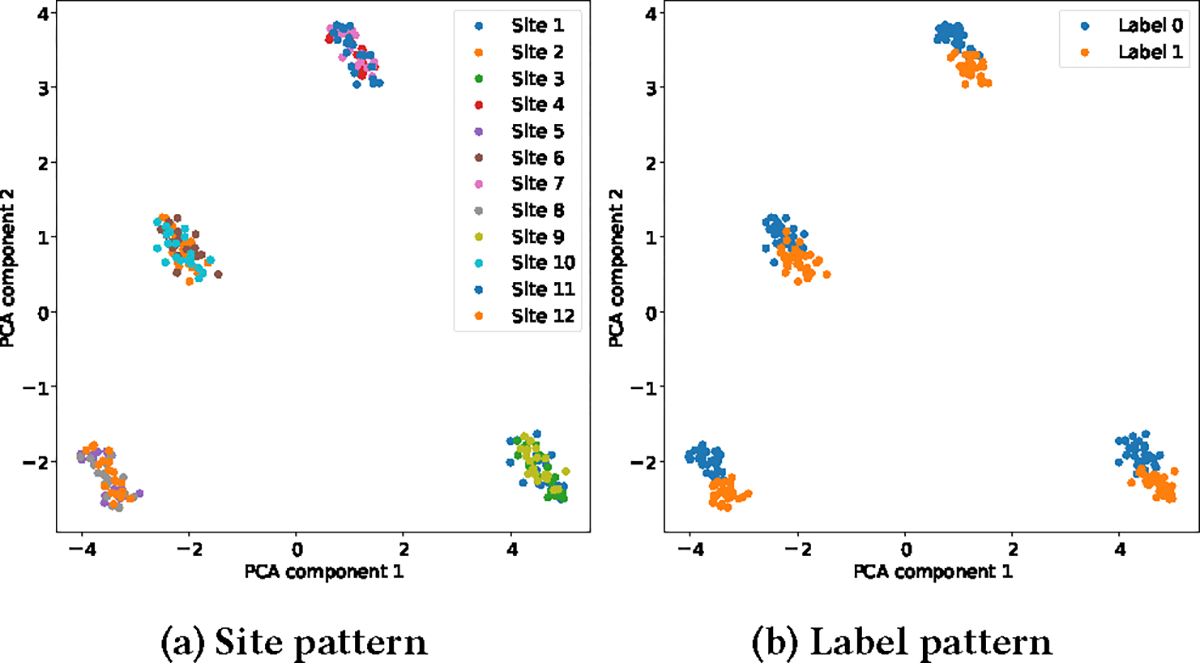
Synthetic Data: site pattern and label pattern of the raw data.

We follow the data generation procedure in [36] and use the graphical model in Figure 1. We replace the site effects with the cluster effects $\gamma_{cg}$. Specifically, the value $y_{ijg}$ with feature index g, site index i, and data point index j is considered as $y_{ijg}∼𝒩\left(\alpha_{g}+\right.\left.X_{ij}\beta_{g}+\gamma_{cg},\delta_{cg}^{2}\sigma_{g}^{2}\right)$. Note that feature values with index g that are from different sites but in the same cluster will be affected by the same cluster effects $\gamma_{cg}$ and $\delta_{cg}$. The ground truth of the harmonized feature for $y_{ijg}$ (expected feature value after harmonization) is $\alpha_{g}+X_{ij}\beta_{g}$. Besides, we induce the task binary label information into the biological covariate $X_{ij}$. For instance, $X_{ij}∼𝒩(0.5,\;0.5)$ and $X_{ij}∼𝒩(-0.5,\;0.5)$ imply positive and negative labels, respectively, so that the ground truth data is linearly separated. To visualize the site pattern, cluster pattern, and label pattern in the synthetic data, Principal Components Analysis (PCA) is employed to reduce the dimension of the data to 2 [24]. Figure 2a and Figure 2b are examples of visualizing the raw (unharmonized) data, which show the patterns of site, cluster, and downstream task labels.

**Algorithm 3:** Distributed Cluster ComBat

**Input:** $y_{ijg}$ - unharmonized data and $X_{ij}$ - biological covariates of sample j from site i
**Output:** ${\overset{ˆ}{\alpha }}_{g}$, ${\overset{ˆ}{\beta }}_{g}$, $\delta_{cg}^{*}$, $\gamma_{cg}^{*}$ - Cluster ComBat parameters and k - trained K-means model
**for all** site i **do**
Estimate ${\overset{ˆ}{\alpha }}_{ig}$, ${\overset{ˆ}{\beta }}_{ig}$, and ${\overset{ˆ}{\gamma }}_{ig}$ locally using least-squared method from data of site i
Send locally estimated ${\overset{ˆ}{\alpha }}_{ig}$, ${\overset{ˆ}{\beta }}_{ig}$, and ${\overset{ˆ}{\gamma }}_{ig}$ to the central server
**end for**
Train K-means model k using ${\overset{ˆ}{\alpha }}_{ig}$, ${\overset{ˆ}{\beta }}_{ig}$, and ${\overset{ˆ}{\gamma }}_{ig}$
Estimate ${\overset{ˆ}{\alpha }}_{g}$, ${\overset{ˆ}{\beta }}_{g}$, and ${\overset{ˆ}{\gamma }}_{ig}$ by taking average of all ${\overset{ˆ}{\alpha }}_{ig}$, ${\overset{ˆ}{\beta }}_{ig}$, and ${\overset{ˆ}{\gamma }}_{ig}$.
**for all** site i **do**
Standardize local data via 2 to get $Z_{ijg}$
Estimate local $Z_{ijg}$
Estimate local $\delta_{ig}^{*}$, $\gamma_{ig}^{*}$ using EmpricalBayes($Z_{ijg}$) via Equation 3
**end for**
**for all** cluster c **do**
Estimate $\delta_{cg}^{*}$, $\gamma_{cg}^{*}$ by taking average of all $\delta_{iq}^{*}$, $\gamma_{ig}^{*}$ for all sites i belong to cluster c.
**end for**
**return** ${\overset{ˆ}{\alpha }}_{g},\;{\overset{ˆ}{\beta }}_{g},\;\delta_{cg}^{*},\;\gamma_{cg}^{*},\;k$

**Algorithm 4:** *Distributed Cluster ComBat* for Unseen Site

**Input:** $y_{ijg}$ - new client’s data, $X_{ij}$ - biological covariate of new client, trained cluster-wise harmonization parameters ${\overset{ˆ}{\alpha }}_{g}$, ${\overset{ˆ}{\beta }}_{g}$, $\delta_{cg}^{*}$, $\gamma_{cg}^{*}$, and trained K-means k
**Output:** $y_{ijg}^{*}$ - harmonized features
Estimate ${\overset{ˆ}{\alpha }}_{ig}$, ${\overset{ˆ}{\beta }}_{ig}$, and ${\overset{ˆ}{\gamma }}_{ig}$ using least-squared method using data from testing client
Get cluster index $\tilde{c}=k({\overset{ˆ}{\alpha }}_{ig},{\overset{ˆ}{\beta }}_{ig},{\overset{ˆ}{\gamma }}_{ig})$
Standardize data via Equation 2
**return** $y_{ijg}^{*}=\frac{{\overset{ˆ}{\sigma }}_{g}}{\delta_{\tilde{c}g}^{*}}(Z_{ijg}-\gamma_{\tilde{c}q}^{*})+{\overset{ˆ}{\alpha }}_{g}+X_{ij}{\overset{ˆ}{\beta }}_{g}$

### 4.2 Synthetic data experiment

**Figure 3:**
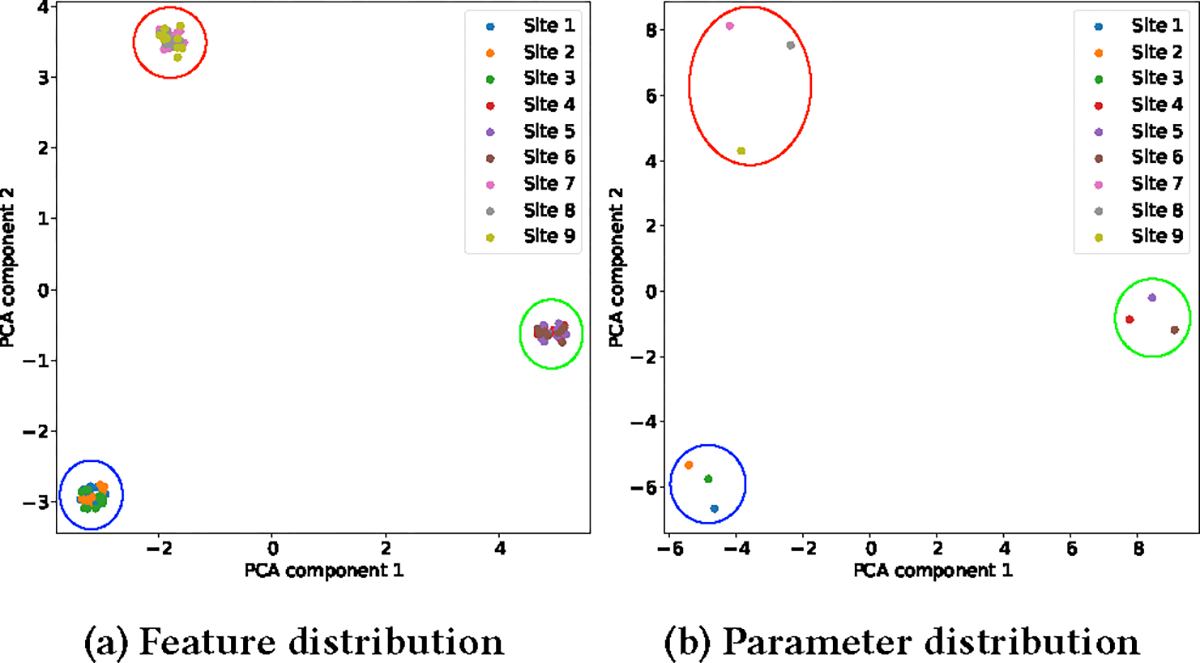
Feature and parameter distribution of synthetic data. Sites within the same cluster in the feature space (as shown in (a)) can also be clustered into the same cluster in the parameter space (as shown in (b)). This indicates that cluster patterns in the feature space can be retained in the parameter space.

We first verify our motivation that if the feature values of sites exhibit cluster patterns in the feature space, then locally estimated feature-wise parameters will also exhibit the same cluster pattern in the parameter space. We generated synthetic data points for 9 sites within 3 clusters for cluster visualization (with data configuration that the number of sites, sample per site, feature, sites per cluster, and biological covariate are 9, 10, 20, 3, and 5 respectively). Specifically, sites 1, 2, and 3 are in the same cluster, sites 4, 5, and 6 are in the same cluster, and sites 7, 8, and 9 are in the same cluster. We used PCA to reduce the dimension to 2 and visualize data points of all sites in the feature space as well as the locally trained parameters for each site in the parameter space. We use colored circles to show the cluster pattern in both feature space and parameter space. As demonstrated in Figure 3, sites within the same cluster in the feature space (as shown in Figure 3a) can also be clustered into the same cluster in the parameter space (as shown in Figure 3b). This indicates that cluster patterns in the feature space can be retained in the parameter space, which verifies our motivation.

Then, we verify the efficacy of our algorithm over COMBAT in both centralized and distributed settings using synthetic data. We generate five synthetic data sets, which follow the graphical model Figure 1 with different parameter configurations. The detailed generation configurations are summarized in Table 1. We assess the performance of *Cluster ComBat* harmonization and original COMBAT algorithms on the synthetic data with aforementioned conditions over two tasks: ground-truth data, i.e., $\alpha_{g}+X_{ij}\beta_{g}$, reconstruction task and ground-truth label classification tasks. Specifically, the Root Mean Square Error (RMSE) between the ground-truth data and the harmonized test data is proposed as the performance measure of the reconstruction task. Also, task accuracy is naturally selected as the performance measure of the downstream classification task. Because the original COMBAT and Distributed ComBat cannot harmonize data from unseen sites, we will retrain harmonization parameters whenever they have data from a testing site. Meanwhile, our proposed *Cluster ComBat* and *Distributed Cluster ComBat* can harmonize testing data without the need for retraining the COMBAT algorithm. In the experiment, we divided the synthetic data into 70% for training and 30% for testing for each task and reported the mean and variance of performance measures over 30 random seeds. The results are summarized in Table 2. The results show that *Cluster ComBat* and *Distributed Cluster ComBat* outperform COMBAT and Distributed ComBat in both tasks over various data conditions.

**Figure 4:**
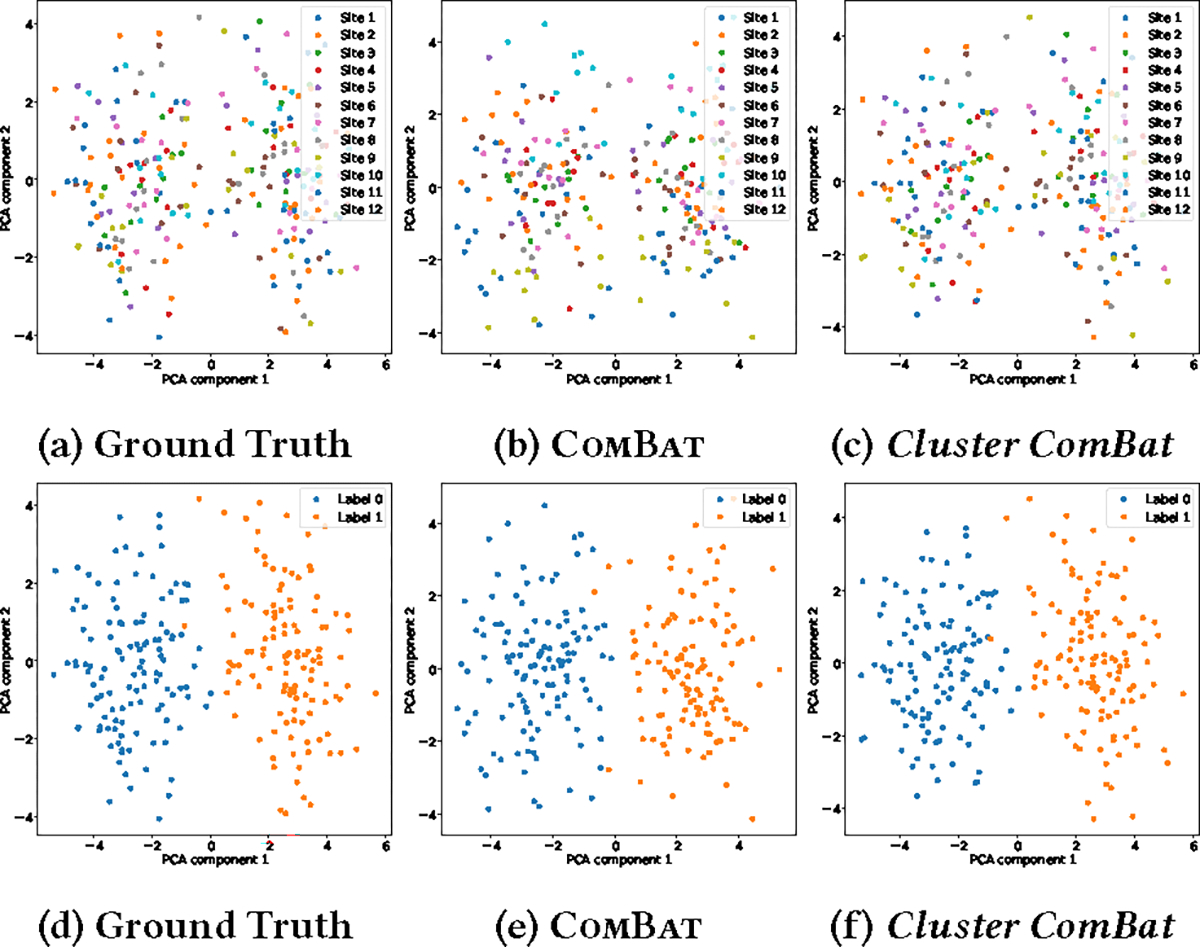
Synthetic Data: site pattern (Figure 4a, 4b, 4c) and label pattern (Figure 4d, 4e, 4f) after harmonization.

Besides, Figure 4 is an example of demonstrating the site pattern and cluster pattern after harmonization (with data configuration that the number of sites, sample per site, feature, sites per cluster, and biological covariate are 12, 20, 20, 3 and 10 respectively). We see that both harmonization methods maintain the task label information, but the site information has been largely erased. We want to reiterate that the original COMBAT, both in centralized or decentralized settings, requires the retraining procedure when harmonizing the testing data. On the contrary, the proposed *Cluster ComBat* eliminates the requirement, benefiting from our parameter-free cluster procedure on unseen data.

## 5 VALIDATION ON BRAIN IMAGING

### 5.1 ADNI Data

We use neuroimaging data from the second phase of the North American Alzheimer’s Disease Neuroimaging Initiative (ADNI) to evaluate our proposed methods. The ADNI data we used has MRI imaging of 563 scans/subjects collected from 18 participating sites. We extracted regional measures from DTI data, following the procedure in [27], leading to 228 features from each scan. We also construct a set of downstream prediction tasks, including the prediction of a set of ADNI-defined indicators derived from the neuropsychological battery to characterize memory, executive function, and language. Specifically: 1) MEM: The ADNI-Mem composite score for memory, which is based on the Rey Auditory Verbal Learning task, word list learning and recognition tasks from ADAS-Cog, recall from Logical Memory I of the Wechsler Memory Test–Revised, and the 3-word recall item from the MMSE [7]. 2) EXF: ADNI-EF composite score for executive function, including Category Fluency (i.e., animals and vegetables), Trail-Making Test parts A and B, Digit Span Backwards, Wechsler Adult Intelligence Scale–Revised Digit–Symbol Substitution, and 5 Clock Drawing items [13]. 3) LAN: ADNI-Lan indicator, which is a composite measure of language [13]. We also include changes in these scores from baselines [16], denoted by MEM SLOPES, EXF SLOPES, and LAN SLOPES, respectively. Later, we use these six target variables to evaluate regression performance in downstream tasks. The characteristic distribution of the ADNI dataset is illustrated in Table 3.

### 5.2 Site and Cluster Effects in Brain Imaging

We first show that site effect and cluster effect do exist in ADNI imaging data. We perform two classification tasks on brain imaging: i) site classification, and ii) cluster classification. For both tasks, the inputs are the raw feature values of the brain imaging samples, and the output labels are the site index for the site classification task and the cluster index for the cluster classification task. We show that harmonization (both COMBAT and *Cluster ComBat*) makes it difficult to distinguish samples from different sites/clusters, i.e., lower site/cluster classification accuracy after harmonization.

For site classification, the site index is a sample’s natural site index, and the overall class number is 18. For cluster classification, we perform K-means to cluster 18 sites into 5 clusters to assign cluster indexes, and thus the overall class number is 5. Specifically, samples with the same cluster indexes can come from the same site or different sites, while samples with different cluster indexes must come from different sites. Logistic Regression is used for both tasks to classify brain imaging. For the train/test split of both tasks, we randomly select 70% of brain imaging as the training set and the remaining 30% for the testing set. The test accuracy results of both tasks are averaged over 100 runs with different random seeds.

Table 4 shows that logistic regression achieves high test accuracy on unharmonized DTI imaging for both tasks. By applying either COMBAT or *Cluster ComBat* harmonization, the test accuracy drops significantly, indicating that either harmonization method makes it harder for the classifier to distinguish between different sites/clusters. This shows that both site/cluster effects on real brain imaging and harmonization methods can alleviate these effects. We also notice that *Cluster ComBat* has higher accuracy compared with COMBAT in site classification with similar accuracy in cluster classification. This can be explained as that after removing cluster effect based on cluster-wise harmonization parameters, differences between clusters are removed by *Cluster ComBat*, while site differences still exist among sites within the same cluster. Thus, it is still possible to differentiate between sites within each cluster even after harmonization in *Cluster ComBat* case. This shows that our assumption for cluster-wise harmonization works well on real brain imaging data.

**Figure 5:**
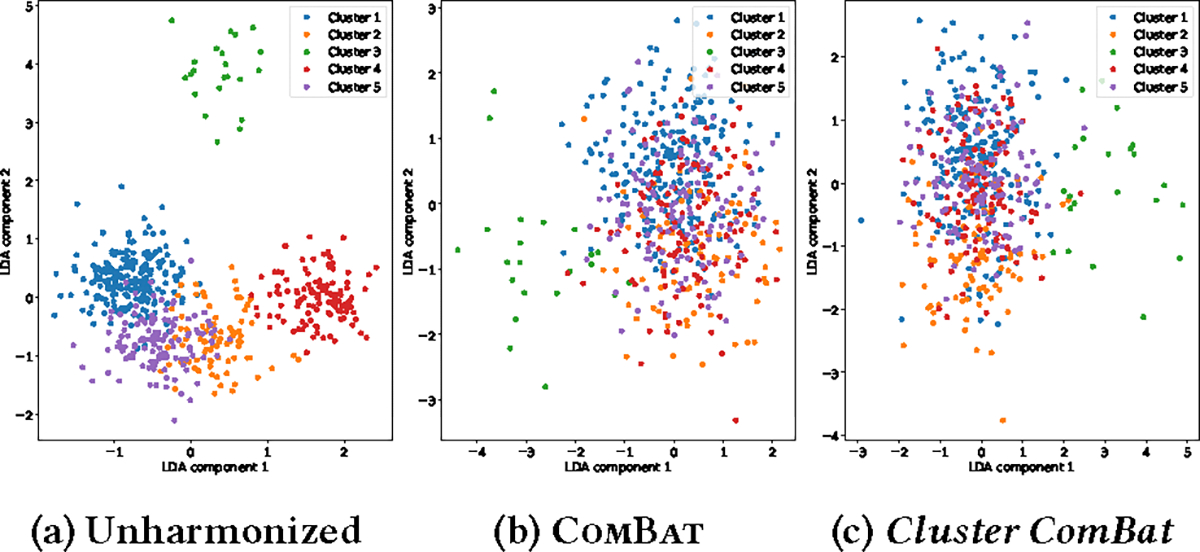
LDA plot of brain imaging data by cluster index

**Figure 6:**
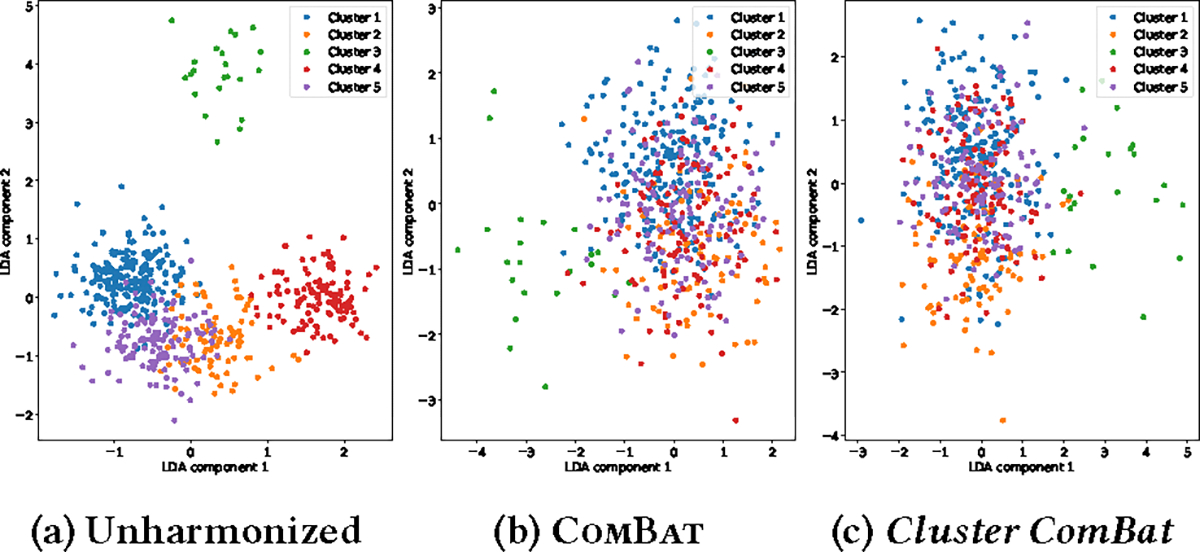
**LDA plot of brain imaging data by site index. Cluster 1 consists of site 3, 6 and 9. Cluster 4 consists of site 1 and 8.** ^[*b*]^ colored by site index, ^[*c*]^ colored by cluster index

Furthermore, we visualize the distributions of DTI imaging features with or without harmonization. We perform the supervised dimension reduction technique Linear Discriminant Analysis (LDA) using site/cluster index as the target variable to reduce 228-dim DTI features to a lower dimensional space with only 2 dimensions. Figure 6 presents the result using site index as the target variable for site effect visualization, and Figure 5 presents the result using cluster index as the target variable for cluster effect visualization.

For cluster effect visualization in Figure 5, we only colored data samples by cluster index. As shown in Figure 5a, data without harmonization reveals a clear distinguishable cluster pattern, especially for cluster 3 and cluster 4, and samples of each cluster are centered around their own cluster centroid. This indicates that cluster effect does exist in DTI imaging. In both Figure 5b and 5c, the distribution of samples presents more like a single spherical shape, and different clusters overlap with each other after harmonization, which makes it harder to distinguish one from others compared with the unharmonized result. This suggests that harmonization methods effectively removed the cluster effect from raw DTI data.

For site effect visualization, we only show distributions of cluster 1 and cluster 4 for demonstration. And we color the same siteindex-based LDA visualization ^1^ using different coloring strategies, for a better understanding of relations between site and cluster effect in *Cluster ComBat*: the left column figures (Figure 6a, 6c) are colored by site index, while the right column figures (Figure 6b, 6d) are colored by cluster index. By comparing Figure 6a and 6b, we can know that both site effect and cluster effect are evident in unharmonized data, as distinct separation is observed between sites and clusters. By comparing Figure 6b and 6d, we verify that our *Cluster ComBat* does remove cluster effect, as cluster 1 and cluster 4 overlap with each other after harmonization. Then, by coloring samples in the same cluster differently based on site index, as shown in Figure 6c, we find that cluster 1 consists of site 3, 6, 9 and 12. Though site 6 and 12 overlap with each other, site 3, 6 and 9 are clearly separated from each other. Similar to cluster 4, site 1 and 8 show obvious disparity with each other. To conclude, our *Cluster ComBat* removes differences over clusters while preserving possible site differences within the cluster, which is also verified in higher site classification accuracy than COMBAT in Table 4.

### 5.3 Downstream Regression Performance

For real data, we do not have the ground truth of harmonization, so our focus is on evaluating the performance of harmonization algorithms through downstream tasks. In these tasks, we use the 228 features of DTI brain imaging to predict the MEM, MEM SLOPES, EXF, EXF SLOPES, LAN, and LAN SLOPES variables. We build a simple Linear Regression model using the Scikit-Learn library [29] to train the regression task on the target target variables. For COMBAT and Distributed ComBat, we retrain parameters as described in Section 4.2. We split the 18 sites into 12 training sites and 6 testing sites, then run experiments 100 times with different combinations of train and test sites. To evaluate performance, we compute the Mean Absolute Error (MAE) of the linear regression’s outputs on the testing site’s data and target testing labels. In a centralized setting, we also compared our method with the Generalized Linear Squares Approach [41], an algorithm designed to eliminate confounding effects. This approach assumes that a variable may be linearly dependent on the confounding variables, and these effects can be removed by solving a linear regression optimization problem. Results in Table 5 show that our proposed method performs better than COMBAT and Generalized Linear Squares Approach in a centralized setting and Distributed ComBat in a decentralized setting for most downstream tasks.

### 5.4 Additional Empirical Studies

#### Time complexity efficiency.

To demonstrate that our proposed *Cluster ComBat* does show better time efficiency compared with COMBAT in both centralized and decentralized settings, we provide an empirical comparison of computation time. We evaluated the average running time (in seconds) for predicting MEM regression results using the ADNI dataset in 100 experiments. As shown in Table 7, *Cluster ComBat* consistently outperforms the original COMBAT in terms of running time, 2× faster in the centralized setting and 4× faster in the decentralized setting.

#### Number of Clusters.

We investigate the impact of the number of clusters (k) for K-means on both *Cluster ComBat* and *Distributed Cluster ComBat*. We conduct the same downstream tasks experiments as described in Section 5.3 with different numbers of clusters for the K-means algorithm, specifically 3, 5, 7, and 9. Average performances are reported in Table 6. As observed in Table 6, variations in the number of clusters (k) do not significantly affect the regression performance across 100 different random seed experiments for all six target variables. This indicates that our *Cluster ComBat* methods are stable among different numbers of clusters.

#### Limited Sample Size Per Sites.

One advantage of our proposed methods is that they can still harmonize data even in limited sample sizes at each site. This is attributed to the fact that we have larger samples in clusters instead of individual sites. We investigated this by restricting the selection to a maximum number of samples at each site, such as 10, 20, 40, 60. We performed a regression task over the EXF variable, and the average performance of 100 experiments is reported in Table 8. We see that when the sample size is limited to 10, COMBAT fails to harmonize. However, our proposed *Cluster ComBat* still achieves comparable regression performance without harmonization. For maximum sample sizes per site of 20, 40, 60, our proposed method consistently outperforms the baseline COMBAT.

#### Important Feature Before and After Harmonization.

For regression tasks, we compute *p*-values for linear regression across 228 features in DTI imaging. The final *p*-values are obtained by averaging over 100 different random seeds. A feature is important if its *p*-value is less than 0.05. Table 9 displays the number of important features for the linear regression across 3 target variables MEM, EXF, and LAN. The table indicates that by using *Cluster ComBat*, we achieve comparable performance with fewer significant features.. This suggests that without harmonization and ComBat, the model may have included too many false positive features.

**Figure 7:**
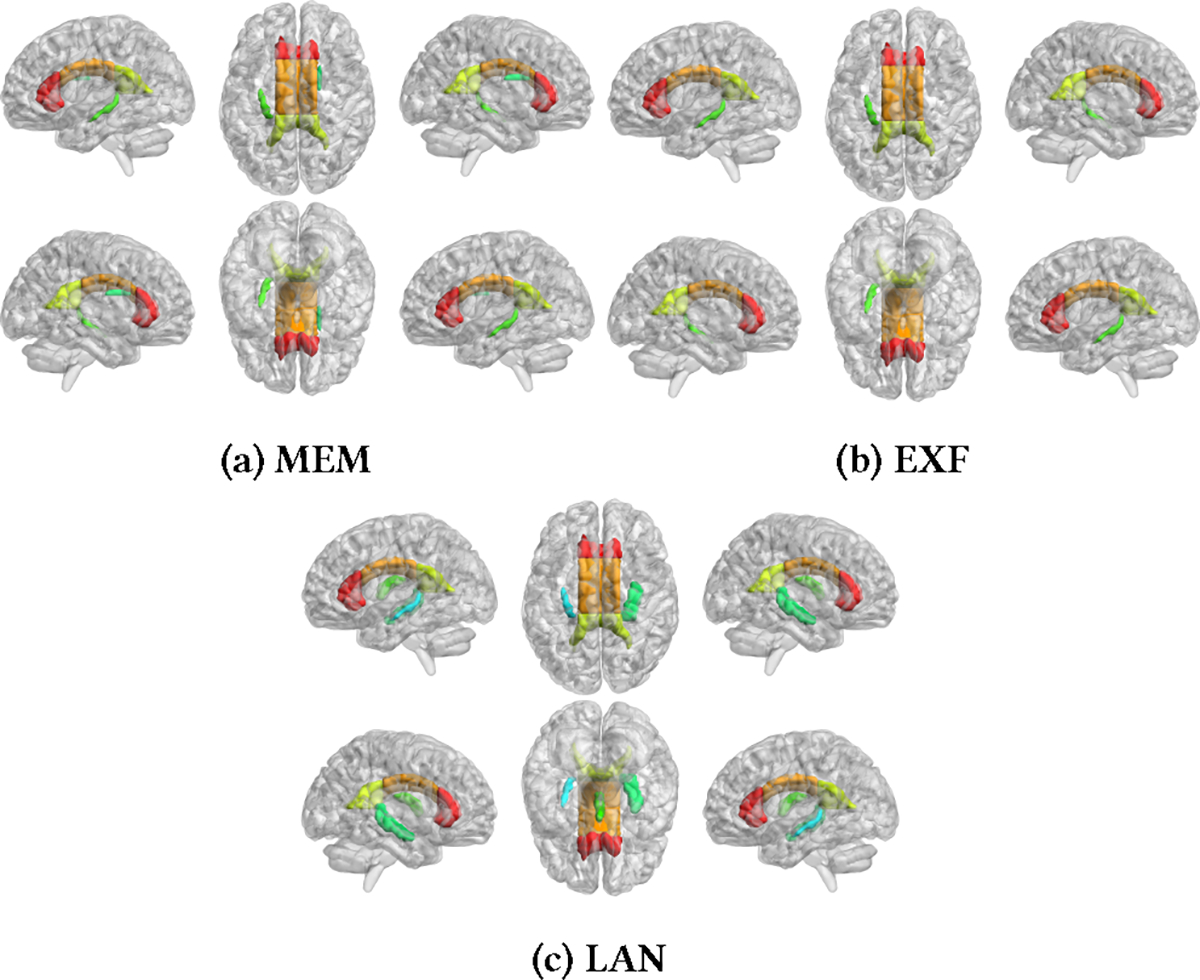
Important Feature Visualization. The left fornix (cres)-stria terminalis and the right superior fronto-occipital fasciculus play a role in MEM. In EXF, the involvement includes the left fornix (cres)-stria terminalis and the full corpus callosum on both sides. For LAN, the engagement extends to the bilateral fornix, full corpus callosum, and bilateral fornix (cres)-stria terminalis. The color in the figure serves solely to distinguish the Regions of Interest (ROIs).

In addition to *p*-values, another measure of feature importance is provided by the linear regression coefficients. The magnitude of the coefficient indicates the importance of a feature. Similar to the approach used for deriving final *p*-values, we compute the average of linear regression coefficients across experiments. Our findings highlight the significant involvement of multiple fiber tracts, such as the fornix(cres)-stria terminalis, superior fronto-occipital fasciculus, corpus callosum, and fornix, in three cognitive tasks: MEM (memory), LAN (language), and EXF (emotion), which are consistent with existing literature [5, 10, 20, 23, 35, 50]. Figure 7 visualizes the details of fiber tracts for each cognitive task.

## 6 DISCUSSION AND CONCLUSION

COMBAT has been the standard protocol for harmonization batch effects for various biomedical data analyses, and yet current COMBAT implementations and variants cannot handle new/unseen sites, once the harmonization is done. In this paper, we developed a novel *Cluster ComBat* and a distributed variant *Distributed Cluster ComBat* to perform privacy-aware harmonization over distributed data sources and handle generalization to data in unseen sites/institutions after the harmonization is completed. Our proposed approach is largely aligned with existing harmonization protocols and can be easily adapted to extend harmonization to large-scale, multi-site data analyses and greatly reduce the logistic overhead of initiating distributed computing when new sites continuously join analyses. We believe this approach can greatly advance data-driven scientific research in multi-institutional studies, especially in the medical and biomedical domains. For example, the research activities [34] in ENIGMA Neuroimaging Consortium [38] can greatly benefit from this research when new institutions join the consortium and participate in existing studies.

We conducted extensive validation on both synthetic data real brain imaging data from ADNI in both centralized and decentralized settings. We demonstrated through both qualitative and quantitative studies that our methods effectively remove cluster-wise effects from brain imaging data. Then, our methods exhibit superior performance on downstream regression tasks compared to baseline harmonization methods in both centralized and decentralized settings, which further validates the efficacy of our harmonized data. We also showed that our methods can use much fewer significant features to achieve similar regression performance compared with other baselines, suggesting potential avenues for further research on selected features.

Regarding deploying our proposed method, we consider 3 implementation consideration aspects: 1) *ML-framework agnostic:* Our algorithm doesn’t involve any specific ML frameworks in the local computation part, so it is easy to implement in many systems regardless of the local ML framework. Flower [4] can be a candidate choice. For downstream tasks after harmonization, like regression or other ML models, the choice of local ML framework can be flexible depending on local preference. 2) *Security communication:* Designed for medical records, the deployment system needs to have communication security to prevent privacy leakage. One possible choice is to encrypt the communication between the clients and the server, for example, Secure Socket Layer (SSL) [15] or Transport Layer Security (TLS) [19]. 3) *Scalable and light-weight:* Since our algorithm’s main benefit lies in new clients joining the federated system, the system deployment should be scalable. To be more specific, when new clients join in, there should be minimum system configuration modification on the server as well as for old clients. Also, the implementation of our algorithm needs to be lightweight, and the FL system with our algorithm should require limited system consumption. And the design of FedLab [51] can be a reference to meet these requirements. As a future work, we will deploy our proposed *Cluster ComBat* harmonization in the ENIGMA Consortium toolbox to further validate existing studies.

## Figures and Tables

**Table 1:** Detailed configurations of synthetic data for simulation

Synthetic Data Index	1	2	3	4	5

#Sites	20	25	30	35	40
#Samples Per Site	20	25	30	35	40
#Features	20	25	30	40	50
#Sites Per Cluster	5	5	5	5	5
#Biological Covariates	5	5	5	5	5

**Table 2:** Validate proposed method using synthetic data.

Algorithm	Performance over different synthetic data conditions									
	Data Reconstruction Task (RMSE)					Data Downstream Task (Acc.)				

	Data-1	Data-2	Data-3	Data-4	Data-5	Data-1	Data-2	Data-3	Data-4	Data-5

Centralized Setting										

Without harmonization	14.35±0.28	35.65±12.15	22.07±1.07	31.53±9.87	31.67±3.03	96.97±0.02	97.57±0.01	98.42±0.00	98.59±0.00	98.85±0.00
ComBat^[a]^	6.53±0.03	14.49±1.73	7.40±0.08	11.74±0.90	9.16±0.23	96.92±0.02	90.10±0.14	98.57±0.00	98.65±0.00	**99.00±0.00**
*Cluster ComBat*	**6.43±0.05**	**14.38±2.75**	**7.29±0.12**	**11.70±1.22**	**9.06±0.32**	**97.03±0.01**	**97.93±0.00**	**98.59±0.00**	**98.84±0.00**	98.94±0.00

Decentralized Setting										

Distributed ComBat^[a]^	6.60±0.03	14.54±1.58	7.42±0.07	11.75±0.84	9.19±0.21	95.53±0.04	90.65±0.27	97.47±0.01	98.02±0.01	97.35±0.03
*Distributed Cluster ComBat*	**6.44±0.05**	**14.37±2.62**	**7.28±0.11**	**11.69±1.17**	**9.04±0.31**	**97.22±0.02**	**97.70±0.01**	**98.68±0.00**	**98.77±0.00**	**98.93±0.00**

[a] means retraining with test sites.

**Table 3:** Characteristic Distribution of ADNI dataset

Variable	All (n = 563)	NL (n = 178)	MCI (n = 292)	AD (n = 93)

Age	75.06±7.28	75.72±6.70	74.44±7.40	75.74±7.81
Gender (%women)	41.39	44.94	41.44	34.41
#Samples Per Site	31.28±19.28	9.89±9.38	16.22±10.50	5.17±5.96
MEM	0.24±0.74	0.86±0.53	0.22±0.45	−0.85±0.45
MEM SLOPES	−0.09±0.11	−0.04±0.06	−0.07±0.08	−0.25±0.09
EXF	0.45±0.62	0.78±0.51	0.47±0.47	−0.25±0.66
EXF SLOPES	−0.06±0.08	−0.03±0.05	−0.05±0.08	−0.13±0.08
LAN	0.45±0.67	0.85±0.44	0.43±0.53	−0.26±0.79
LAN SLOPES	−0.07±0.09	−0.03±0.05	−0.06±0.07	−0.18±0.10

**Table 4:** Accuracy of site and cluster classification on brain imaging data

Harmonization Algorithm	Site	Cluster

Without harmonization	86.98±0.078	82.23±0.067
*Cluster ComBat*	36.70±0.091	19.32±0.073
ComBat	6.93±0.034	20.75±0.076

**Table 5:** Performance of downstream regression task for neuroimaging dataset.

Algorithm	MEM	MEM SLOPES	EXF	EXF SLOPES	LAN	LAN SLOPES

Centralized Setting						

Without harmonization	13.77±22.05	1.89±3.59	10.30±19.38	1.58±3.19	10.94±17.74	1.45±3.01
Generalized Linear Squares Approach [41]	1.07±0.30	0.52±0.18	0.93±0.22	0.47±0.18	0.95±0.26	0.45±0.13
ComBat^[a]^	**1.00 ±0.18**	0.16±0.04	1.03±0.18	0.13±0.04	1.04±0.20	0.13±0.03
*Cluster ComBat*	1.00±0.20	**0.15±0.03**	**0.91±0.12**	**0.12±0.03**	**0.87±0.15**	**0.12±0.02**

Decentralized Setting						

Distributed ComBat^[a]^	0.98±0.16	0.15±0.03	1.00±0.16	0.13±0.03	1.01±0.17	0.12±0.03
Distributed *Cluster ComBat*	**0.91±0.16**	**0.14±0.03**	**0.96±0.12**	**0.12±0.02**	**0.91±0.17**	**0.11±0.02**

[a] means retraining with test sites.

**Table 6:** Effect of number of clusters *k* on *Cluster ComBat* for downstream regression task

k	MEM	MEM SLOPES	EXF	EXF SLOPES	LAN	LAN SLOPES
			(Centralized setting / Decentralized setting)			

3	1.06±0.35 / 0.91±0.16	0.16±0.07 / 0.14±0.03	0.93±0.25 / 0.96±0.12	0.13±0.03 / 0.12±0.02	0.93±0.23 / 0.91±0.17	0.13±0.05 / 0.11±0.02
5	1.00±0.20 / 0.93±0.16	0.15±0.03 / 0.14±0.03	0.91±0.12 / 0.97±0.13	0.12±0.03 / 0.12±0.02	0.87±0.15 / 0.90±0.14	0.12±0.02 / 0.12±0.02
7	1.02±0.24 / 0.96±0.17	0.16±0.04 / 0.14±0.03	0.92±0.22 / 0.98±0.13	0.12±0.02 / 0.12±0.02	0.91±0.18 / 0.92±0.14	0.13±0.03 / 0.12±0.02
9	1.07±0.23 / 1.01±0.19	0.17±0.03 / 0.15±0.03	0.94±0.18 / 1.02±0.14	0.13±0.03 / 0.13±0.02	0.93±0.18 / 0.97±0.17	0.14±0.03 / 0.13±0.02

**Table 7:** Time efficiency of harmonization algorithms for MEM regression task.

Algorithm	Average Time (s)

Centralized Setting	

ComBat^[a]^	0.2427±.0.0017
*Cluster ComBat*	0.1127±.0.0001

Decentralized Setting	

Distributed ComBat^[a]^	2.5051±0.0771
Distributed *Cluster ComBat*	0.6389±.0.0027

[a] means retraining with test sites.

**Table 8:** Effect of limiting number of samples *n* per site for harmonization methods.

Algorithm	*n* = 10	*n* = 20	*n* = 40	*n* = 60

Without harmonization	0.73±0.15	33.60±79.37	20.45±39.30	10.84±20.11
ComBat^[a]^	2.03±0.70	2.71±1.09	1.10±0.22	1.06±0.18
*Cluster ComBat*	0.80±0.16	2.36±1.11	0.97±0.28	0.91±0.14

[a] means retraining with test sites.

**Table 9:** Number of important features (*p* < 0.05) for MEM, EXF, LAN regression task.

Algorithm	MEM	EXF	LAN

Without harmonization	150	127	147
ComBat^[a]^	156	135	150
*Cluster ComBat*	26	31	27

[a] means retraining with test sites.

## Box

CCS CONCEPTS

• Applied computing → Imaging; • Computing methodologies → Distributed algorithms; Machine learning.

## Footnotes

1 LDA visualization results will differ depending on the choice of target variable.
